# Cyclic Stretch Alters Vascular Reactivity of Mouse Aortic Segments

**DOI:** 10.3389/fphys.2017.00858

**Published:** 2017-10-30

**Authors:** Arthur Leloup, Sofie De Moudt, Cor Van Hove, Paul Fransen

**Affiliations:** ^1^Laboratory of Physiopharmacology, Department of Pharmaceutical Sciences, University of Antwerp, Antwerp, Belgium; ^2^Laboratory of Pharmacology, Faculty of Medicine and Health Sciences, University of Antwerp, Antwerp, Belgium

**Keywords:** aorta, contraction, isometric, cyclic stretch, relaxation, nitric oxide

## Abstract

Large, elastic arteries buffer the pressure wave originating in the left ventricle and are constantly exposed to higher amplitudes of cyclic stretch (10%) than muscular arteries (2%). As a crucial factor for endothelial and smooth muscle cell function, cyclic stretch has, however, never been studied in *ex vivo* aortic segments of mice. To investigate the effects of cyclic stretch on vaso-reactivity of mouse aortic segments, we used the Rodent Oscillatory Tension Set-up to study Arterial Compliance (ROTSAC). The aortic segments were clamped at frequencies of 6–600 bpm between two variable preloads, thereby mimicking dilation as upon left ventricular systole and recoiling as during diastole. The preloads corresponding to different transmural pressures were chosen to correspond to a low, normal or high amplitude of cyclic stretch. At different time intervals, cyclic stretch was interrupted, the segments were afterloaded and isometric contractions by α_1_-adrenergic stimulation with 2 μM phenylephrine in the absence and presence of 300 μM L-NAME (eNOS inhibitor) and/or 35 μM diltiazem (blocker of voltage-gated Ca^2+^ channels) were measured. As compared with static or cyclic stretch at low amplitude (<10 mN) or low frequency (0.1 Hz), cyclic stretch at physiological amplitude (>10 mN) and frequency (1–10 Hz) caused better *ex vivo* conservation of basal NO release with time after mounting. The relaxation of PE-precontracted segments by addition of ACh to stimulate NO release was unaffected by cyclic stretch. In the absence of basal NO release (hence, presence of L-NAME), physiological in comparison with aberrant cyclic stretch decreased the baseline tension, attenuated the phasic contraction by phenylephrine in the absence of extracellular Ca^2+^ and shifted the smaller tonic contraction more from a voltage-gated Ca^2+^ channel-mediated to a non-selective cation channel-mediated. Data highlight the need of sufficient mechanical activation of endothelial and vascular smooth muscle cells to maintain basal NO release and low intracellular Ca^2+^ in the smooth muscle cells in large arteries. Both phenomena may play a vital role in maintaining the high compliance of large arteries.

## Introduction

In the cardiovascular system large elastic arteries such as the aorta or carotid artery are well equipped to dampen the pressure wave originating from the left ventricle. Because of their close proximity to the left ventricular ejection, elastic arteries are constantly exposed to high levels of cyclic stretch and dilate by approximately 10% with each heartbeat. This cyclic stretch is much higher than in muscular arteries, which dilate by only 2–3% (O'Rourke and Hashimoto, 2007), has a major role in regulating endothelial cell (EC) and vascular smooth muscle cell (VSMC) function, and is essential for the normal development, homeostasis, and remodeling of the vasculature (Chatterjee et al., 2015). Elastic more than muscular arteries are subject to progressive arterial stiffening (Laurent et al., 1994; Ruitenbeek et al., 2008; Borlotti et al., 2012; Zhang et al., 2013), pointing to different regulations of arterial compliance in the different vessel types and a complex interaction between larger and smaller arteries in the development of arterial stiffness and hypertension (Benetos et al., 1993; Laurent et al., 2009). Our recent observations that elastic arteries in mice mainly differ from the smaller muscular arteries by releasing more nitric oxide (NO) in baseline conditions (Leloup et al., 2015b) suggests that basal release of NO may be a key factor in the development of progressive arterial stiffening (van Langen et al., 2012).

Current knowledge on cyclic stretch-dependent EC and VSMC phenotypic modulation originates almost exclusively from *in vitro* data, obtained from cultured cells grown on elastomer-bottomed culture plates (Mantella et al., 2015). For example, in bovine aortic endothelial cells cultured in compliant tubes, PI-3K/Akt signaling and eNOS phosphorylation increased with pulsatile rather than steady flow, which was not the case in cells cultured in stiff tubes (Peng et al., 2003). These data suggest that basal NO release from aortic segments might be dependent on cyclic stretch. Likewise in VSMC, cyclic stretch has been shown to be essential in maintaining their contractile phenotype, which can be illustrated by the “arterialization” of venous blood vessels used in coronary artery bypass surgery, where the orientation and number of VSMC cells in the medial layer adapts as a result of their exposure to arterial pressures (Thorin-Trescases and Thorin, 2016). In general, the *in vitro* data on the effects of cyclic stretch on EC as well as VSMC have not led to coherent conclusions, possibly because they lack the cross-talk between EC and VSMC, and their extracellular matrix, which are of important physiological relevance. At least, physiological cyclic stretch exposure of *ex vivo* pig carotid artery has been demonstrated to support a differentiated and fully functional phenotype of VSMC, whereas reduced stretch caused endothelial dysfunction and oxidative stress (Gambillara et al., 2008; Thacher et al., 2009, 2010).

Cyclic stretch of the elastic arteries can display enormous fluctuations in frequency and amplitude, for example during physical activity or in hypertension. In the present study, we subjected aortic segments to a range of cyclic stretch amplitudes and frequencies for 1–4 h to investigate “acute” effects on EC and VSMC function. Therefore, isolated mouse aortic segments were mounted in a new set-up, the Rodent Oscillatory Tension Set-up to study Arterial Compliance (ROTSAC), which allowed for the stretching of *ex vivo* mouse aortic segments, with an intact cross-talk between EC, VSMC and the extracellular matrix (Leloup et al., 2016). The present study hypothesized that cyclic stretch exposure of aortic segments at physiological amplitudes and frequencies can alter the contractile properties of these segments, which are usually studied in isometric conditions only.

## Methods

### Animals

Male C57Bl6 mice (*n* = 39, 4–9 months, weight ranging from 28.5 ± 0.7 g at 4 months to 33.7 ± 0.9 g at 9 months) were housed in the animal facility of the University of Antwerp in standard cages with 12–12 h light-dark cycles and free access to regular chow and tap water. Animals were euthanized by perforating the diaphragm while under anesthesia (sodium pentobarbital, Sanofi, Belgium, 75 mg kg^−1^, i.p.). The thoracic aorta was carefully removed and stripped of adherent tissue. Starting at the diaphragm, the aorta was cut in 4 segments of 2 mm width. Segments were immersed in Krebs Ringer (KR) solution (37°C, 95% O_2_/5% CO_2_, pH 7.4) containing (in mM): NaCl 118, KCl 4.7, CaCl_2_ 2.5, KH_2_PO_4_ 1.2, MgSO_4_ 1.2, NaHCO_3_ 25, CaEDTA 0.025 and glucose 11.1. The zero external Ca^2+^ solution had the same composition except that 2.5 mM CaCl_2_ was omitted and 1 mM EGTA was added. To normalize external Ca^2+^, 3.5 mM CaCl_2_ was added to the zero calcium solution. To avoid any vasomotor interference due to prostanoids, 10 μM indomethacin (Federa, Belgium) was present in all experiments. The studies were approved by the Ethical Committee of the University of Antwerp (2015–52 and 2016–28), and all experiments were performed conform to the Guide for the Care and Use of Laboratory Animals published by the US National Institutes of Health (NIH Publication No. 85–23, revised 1996).

### ROTSAC

**Figure 1:**
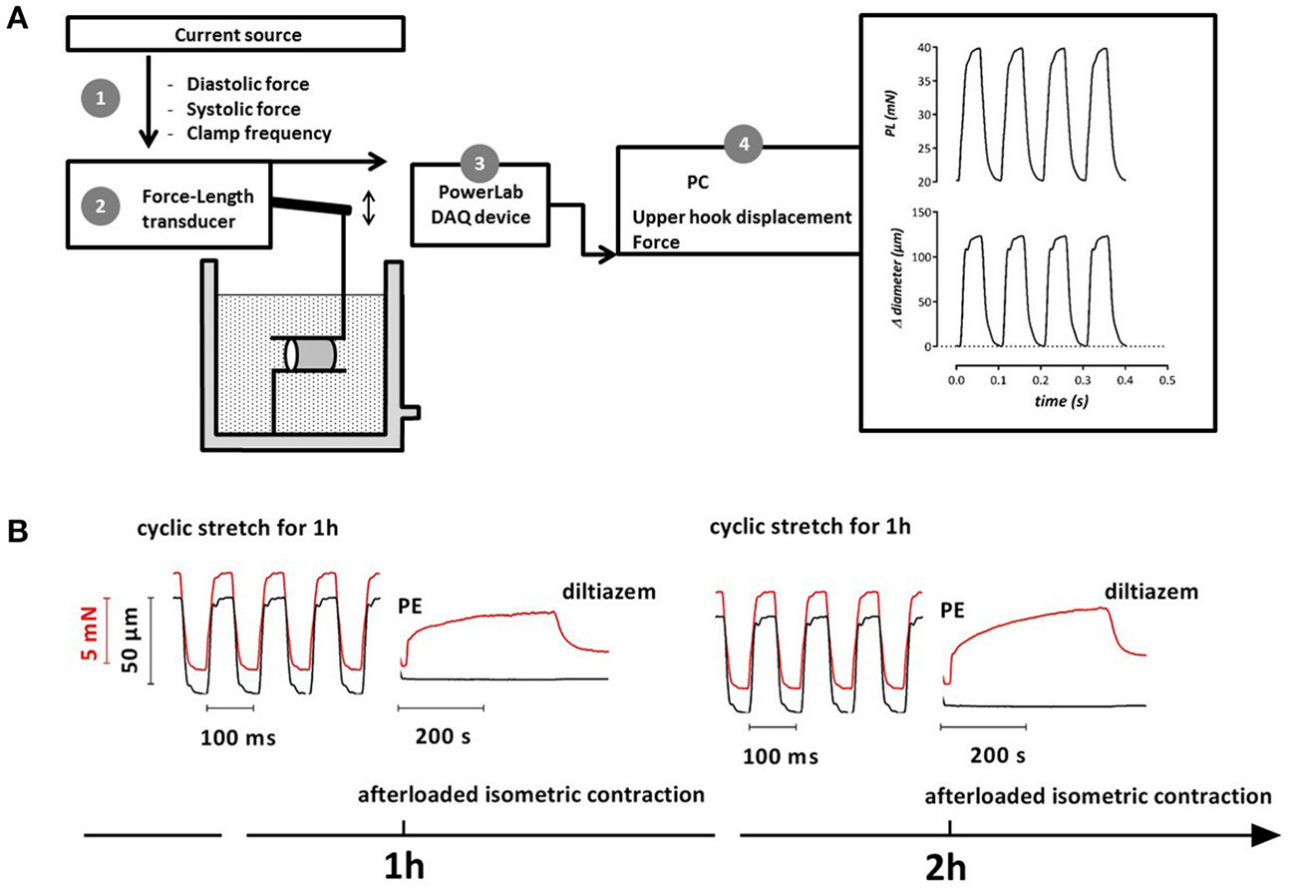
Schematic diagram of the ROTSAC **(A)** (Leloup et al., 2016) and example of experimental protocol **(B)**. After mounting the aortic segments a current source was used to control the distension force and clamp frequency of the force-length transducer via the upper hook (1). Force and displacement were measured by the transducer (2) and acquired at 1 kHz by a PowerLab DAQ device (3). Diameter, length and force were used to calculate the pressure that would exist in an equilibrated vessel segment with the given dimensions and wall stress (4). **(B)** Example (1 segment of 1 mouse) of tracings for an experimental protocol to determine vascular reactivity of aortic segments subjected to variable periods of cyclic stretch. Segments were stretched at a frequency of 10 Hz to alternating preloads (red, 20–28 mN in this condition). The corresponding diameter change is shown in black. After 1 h cyclic stretch, the segment was afterloaded to measure the isometric contraction induced by 2 μM PE and subsequent relaxation by 35 μM diltiazem. The same measurements could be performed at later time points (2 h, 3 h, or later).

After isolation of the thoracic aorta, the 4 segments were each mounted in the new set-up (ROTSAC, Figure 1A) between two wire hooks connected to a force-displacement transducer that is set to oscillate between two experimental preloads at a certain frequency allowing us to control the level of cyclic stretch (Leloup et al., 2016). The alternating preloads are expressed in mN, the corresponding transmural pressures can be estimated by applying Laplace's law. Assuming a thin, isotropic and homologous wall for large vessels such as the aorta, the transmural pressure (P, in mm Hg) is directly proportional to the preload (PL, in mN) and inversely proportional to its length (l, in mm) and radius (r, in mm): $=\frac{PL}{l^{*}2r}*7.5\text{ mmHg}/\text{kPa}$. During a cyclic stretch period the segments' extrapolated diameter and preload are continuously measured at an acquisition rate of 1 kHz (Powerlab, Labchart).

#### Different amplitudes of applied stretch

**Figure 2:**
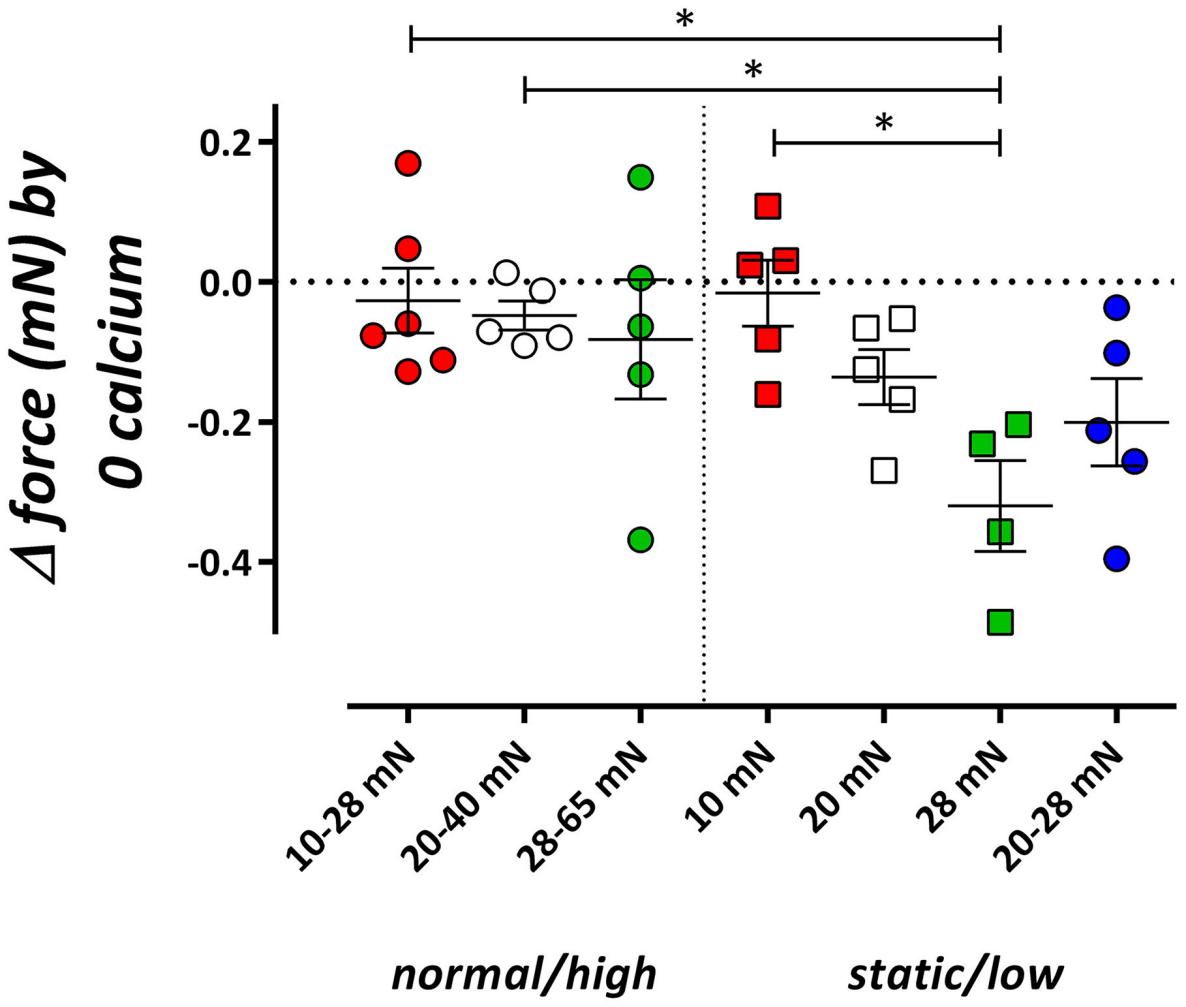
Influence of cyclic stretch conditioning on isometric force upon Ca^2+^ removal from the extracellular solution. Isometric force was measured at 10, 20, and 28 mN in segments, which were previously conditioned at 10–28, 20–40, or 28–65 mN cyclic stretch (normal/high) or which were continuously preloaded at 10, 20, and 28 mN or stretched at low amplitude (20–28 mN) (static/low). ^*^P < 0.05, *n* = 4–6, one-way ANOVA with Tukey's multiple comparison test.

Aortic segments were stretched at frequencies of 600/min (10 Hz) with damped clamps of 50 ms to each pressure (see Figures 1, 2). The preloads were chosen to mimic low, normal or high cyclic stretch at different transmural pressures (Table 1).

**Table 1:** Preloads (PL) and corresponding transmural pressures (BP).

**Cyclic stretch**	**Low**	**Normal**	**Normal**	**High**
Cyclic PL	20–28 mN	10–28 mN	20–40 mN	28–65 mN
Cyclic BP	70–85 mm Hg	50–85 mm Hg	70–120 mm Hg	85–160 mm Hg
Δ diameter	46 ± 2 μm	123 ± 2 μm	126 ± 3 μm	145 ± 4 μm

Cyclic stretch caused diameter changes (Table 1, *n* = 7–9), which were significantly (*p* < 0.001) lower for the 20–28 mN cyclic stretch, which were of comparable magnitude for 10–28 and 20–40 mN and significantly larger (*p* < 0.001) at 28–65 mN. With mean diameters of the mouse aortic segments at 20 mN of 1,060 ± 11 μm (*n* = 10), the cyclic stretch amounted 4, 12, 12, and 14% at 20–28, 10–28, 20–40 and 28–65 mN, respectively.

#### Different frequencies of applied stretch

Segments' clamp frequencies were changed from 10 to 5, 2.5, 1.25, 1, and 0.1 Hz at stretch amplitudes of 20 mN or 50 mm Hg (20–40 mN or 70–120 mm Hg), with damped clamps of 50 ms to 40 mN and variable duration to 20 mN.

#### Experimental protocols

Contractile properties of all the segments mounted in the ROTSAC were always measured in isometric conditions by interrupting the cyclic stretch and applying an afterload of 20 mN (70 mm Hg) to the lower preload at which the segments were clamped (10, 20, and 28 mN or 50, 70, and 85 mm Hg). These contractions were compared with the isometric contractions of the segments, which were continuously conditioned without cyclic stretch. As such, the diameter of all the segments was kept constant at the lower preload for cyclically stretched segments and at its single preload for the statically stretched segments. Different amplitudes of cyclic or static stretch were not applied to a single segment but delivered to different segments. For each experimental day 4 segments of one mouse were mounted in parallel in 4 ROTSAC set-ups. Two segments were stretched to 20–28 and 20–40 mN (cyclic stretch), whereas the two other segments were stretched to 20 and 28 mN (static stretch). The next experimental day two segments were stretched to 10–28 and 28–65 mN (cyclic stretch) whereas static stretch was applied to 2 other segments (10 and 65 mN). The static data at 65 mN are not included in the presented data. These experiments were repeated for 5 or 6 mice for each experimental protocol. After conditioning with cyclic or static stretch, segments were afterloaded with 20 mN, isometric data (see Figure 1B) upon contraction or relaxation were acquired at 10 Hz (Powerlab, Labchart) and compared with isometric contractions performed in organ baths in other studies.

Afterloaded or isometric contraction was elicited by adding the α_1_-adrenoceptor agonist phenylephrine (PE) at a concentration of 2 μM, which is a maximally effective concentration. Both phasic (in the absence of extracellular Ca^2+^) and tonic contractions (after addition of 3.5 mM Ca^2+^) were measured. The PE-induced phasic contraction was measured 3 min after the removal of extracellular Ca^2+^ by switching from normal KR to KR without Ca^2+^ and containing 1 mM EGTA (0 Ca solution) (Leloup et al., 2015a). Recordings of the phasic contractions were analyzed with a double exponential function

$$Y=Aon^{*}(1-\exp\left(-\frac{X-X0}{\tau on}\right)+Aoff^{*}(1-\exp(-\frac{X-X0}{\tau off})$$

to calculate the time constants (τ_on_, τ_off_) and amplitudes (A_on_, A_off_) of contraction and relaxation of these phasic contractions, respectively. PE-induced contractions were also measured in normal, Ca^2+^ containing KR solution and these contractions were also analyzed by a double exponential function revealing time constants (τ_fast_, τ_slow_) and amplitudes (A_fast_, A_slow_) of the fast and slow force component. The fast component refers to the phasic contraction by PE, whereas the slow component corresponds with the contraction due to Ca^2+^ influx (Fransen et al., 2015). Isometric contractions could be determined in the absence or presence of 300 μM L-NAME to block basal eNOS activity and to study effective basal NO release.

To reveal the contribution of Ca^2+^ influx via L-type Ca^2+^ channels to the total contraction, Ca^2+^ influx via voltage-gated L-type Ca^2+^ channels (VGCC) was inhibited with 35 μM diltiazem, causing partial relaxation of the contraction by PE (see Figure 1B). The contraction, remaining after addition of diltiazem, is due to Ca^2+^ influx via non-selective cation channels (NSCC) (Fransen et al., 2015).

To further study the basal release of NO in the different cyclic stretch conditions, contractions by PE were measured at 1 h time intervals after mounting the segments in the ROTSAC (see Figure 1B) and finally by adding 300 μM L-NAME to inhibit eNOS. We have previously shown that the most sensitive way to measure basal NO release is to measure its contraction-depressing effect at different time intervals and at the end of the experiment the NO-independent contraction in the presence of L-NAME (van Langen et al., 2012; Leloup et al., 2015b). Relaxation of segments pre-contracted with 2 μM PE by endogenous NO release was determined by constructing concentration-relaxation curves for acetylcholine (ACh, 3^*^10^−9^ −3^*^10^−6^ M). These concentration-relaxation curves were fitted with sigmoidal concentration-response equations with variable slope (GraphPad Prism) to obtain E_max_- and logEC_50_-values for ACh.

All data are expressed as mean ± SEM (with n, the number of mice). Statistical analysis was performed with Graphpad Prism 6 software. Dependent on the type of experiment, one and two way ANOVA with Tukey's or Dunnett's multiple comparison test was applied to the data to select significance (*P* < 0.05).

## Results

### Static vs. cyclic stretch: focus on VSMC

Aortic segments were conditioned with cyclic stretch of variable amplitude in the presence of 300 μM L-NAME to inhibit basal NO release from the endothelium and to focus on the effects of cyclic stretch on VSMC. Isometric contractions were measured by interrupting the cyclic stretch and stimulating the afterloaded segments with the α_1_-adrenoceptor agonist PE (2 μM). PE-induced phasic contractions were determined in the absence of extracellular Ca^2+^, whereas tonic contractions were measured upon re-addition of external Ca^2+^. The tonic and phasic isometric contractions after normal or high cyclic stretch (10–28, 20–40, and 28–65 mN, further called normal/high) were compared with the contractions of segments which were continuously mounted in low amplitude cyclic stretch (20–28 mN) or static conditions (10, 20, and 28 mN), further called static/low.

#### Phasic contractions by PE

In the static stretch segments, removal of external Ca^2+^ induced a preload-dependent decrease of baseline isometric force (Figure 2, De Moudt et al., 2017), which was absent when segments were previously conditioned with normal/high cyclic stretch. At low amplitude cyclic stretching (20–28 mN) however, removal of external Ca^2+^ tended to decrease preload similarly as in isometric segments.

**Figure 3:**
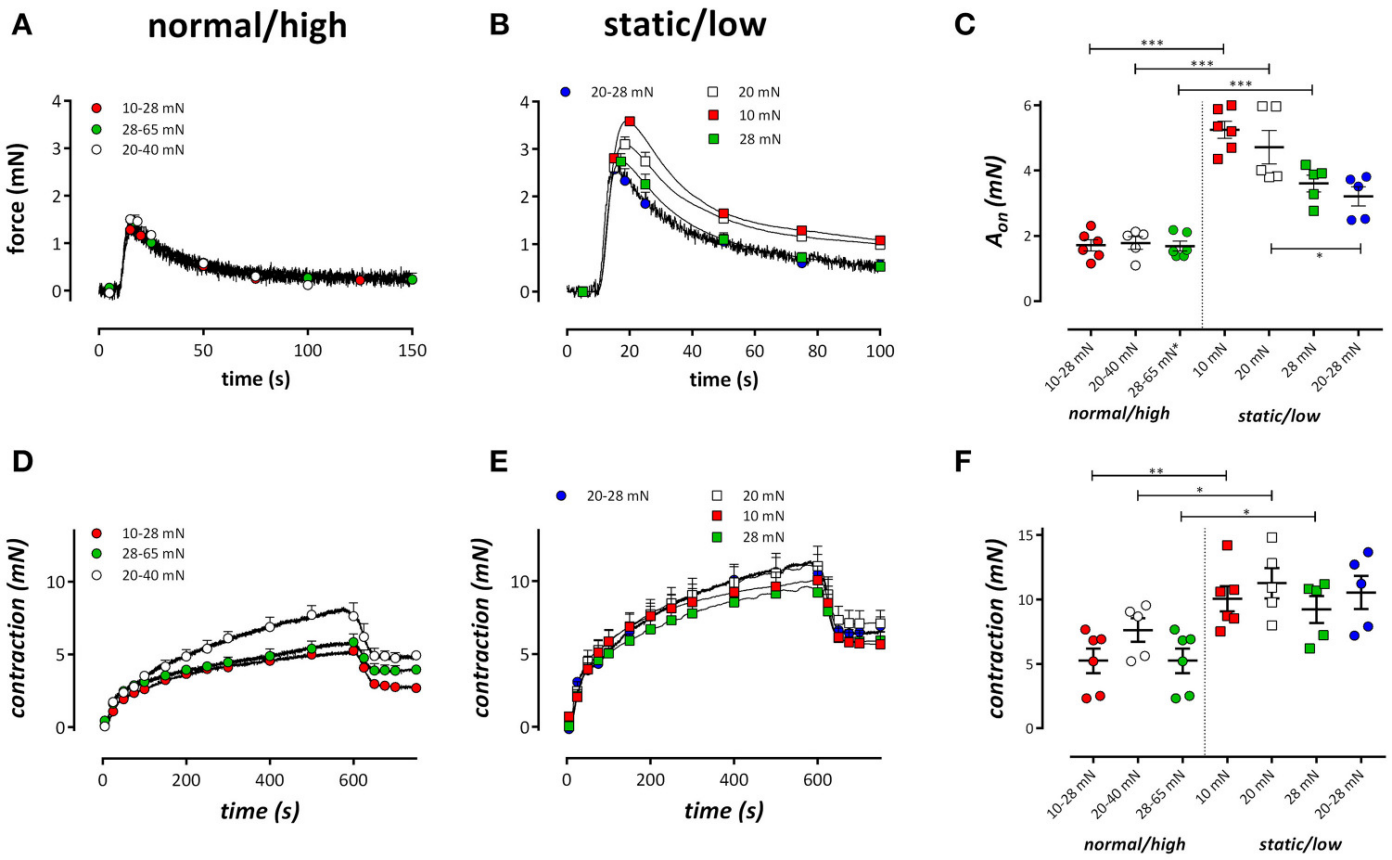
Tonic and phasic contractions by 2 μM PE. Tonic contractions by PE in the absence of external Ca^2+^ in segments conditioned at cyclic stretch of 10–28 (red), 20–40 (white), or 28–65 (green) mN **(A)** or static stretch at 10 (red), 20 (white), or 28 mN (blue) or low amplitude cyclic stretch at 20–28 mN (blue). **(C)** Displays the amplitude of the contraction as revealed by a double exponential fit of the contractions. **(D,E)** Show the phasic contractions in the same conditions as **(A,B)** but after re-admission of extracellular Ca^2+^. At 600 s, the VGCC blocker diltiazem (35 μM) was added to determine the relative amount of VGCC-mediated Ca^2+^ influx to the PE-induced tonic contraction. In **(F)** the contraction by PE after 600 s is plotted for the different conditions. ^*^P < 0.05, ^**^P < 0.01, ^***^P < 0.001 vs. corresponding static or low stretch conditions. One-way ANOVA with Dunnett's multiple comparison test.

After 1 h cyclic stretch at static, low, normal and high stretch conditions in the presence of L-NAME, phasic and tonic contractions were measured in isometric conditions at the preload according to the static stretch or lower cyclic stretch preload of 10, 20, and 28 mN (Figure 3).

α_1_-Adrenoceptor stimulation of mouse aortic segments with 2 μM PE in the absence of external Ca^2+^ caused phasic contractions (Fransen et al., 2015; Leloup et al., 2015a), which decreased with higher preloads in static stretch segments, as described previously (De Moudt et al., 2017; Figures 3A–C). Conditioning of the segments for 1 h with normal/high cyclic stretch amplitude caused smaller IP_3_-mediated contractions by PE. Contractions were analyzed with a double exponential function, revealing time constants (τ_on_, τ_off_) and amplitudes (A_on_, A_off_) of contraction and relaxation of these phasic contractions, respectively. The time constants of contraction or relaxation were not significantly different between the conditions (τ_on_ was 2.1 ± 0.3 s, 1.7 ± 0.2 s, 1.5 ± 0.1 s, 1.5 ± 0.1 s and τ_off_ was 22.7 ± 2.9 s, 25.5 ± 2.6 s, 24.0 ± 3.2 s, 21.5 ± 1.8 s for 10–28, 20–40, 28–65, and 20–28 mN, respectively). Contraction and relaxation amplitudes did not differ between 10–28, 20–40, and 28–65 mN cyclic stretch, but they were all significantly different from A_on_ (Figure 3C) and A_off_ in the low cyclic and static stretch conditions.

#### Tonic contractions by PE

**Figure 4:**
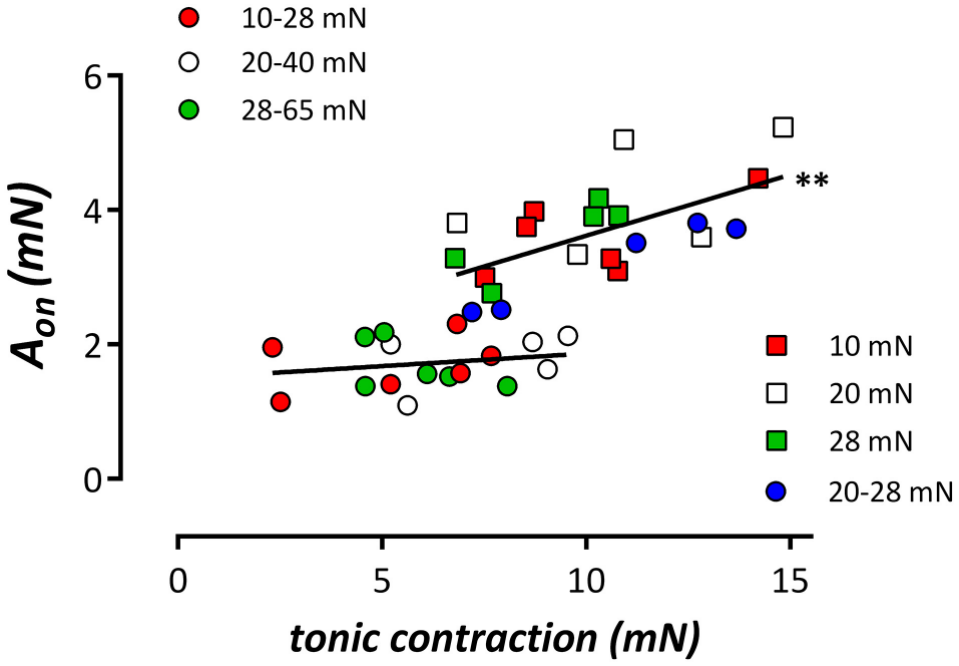
Relationship between the amplitude of the phasic contraction (IP_3_ contraction, A_on_) by 2 μM PE and the contraction induced by re-addition of extracellular Ca^2+^ (tonic contraction) in the different stretch conditions. Linear regression revealed relationships with slopes (full lines), which were non-significantly different from zero for the segments cyclically stretched at normal/high amplitudes, but which were significantly different from zero in all other (low/static stretch) conditions. Both slopes were significantly different (pearson correlation, ^**^P < 0.01).

Following re-addition of extracellular Ca^2+^ to the segments, PE-induced contractions, which are now due to Ca^2+^ influx, were significantly lower at normal/high cyclic stretch than at low cyclic or static stretch (Figures 3D,E). Considering Figures 3C,F, it seemed that the amplitude of the IP_3_-mediated contraction by PE (Figure 3C) was correlated with the total contraction after addition of external Ca^2+^ (Figure 3F). When both parameters were plotted against each other for the different conditions (Figure 4), there was a positive correlation between IP3-mediated contractions and tonic contractions for the low cyclic and static stretch conditions. This correlation was absent for the normal/high cyclic stretch conditions.

**Figure 5:**
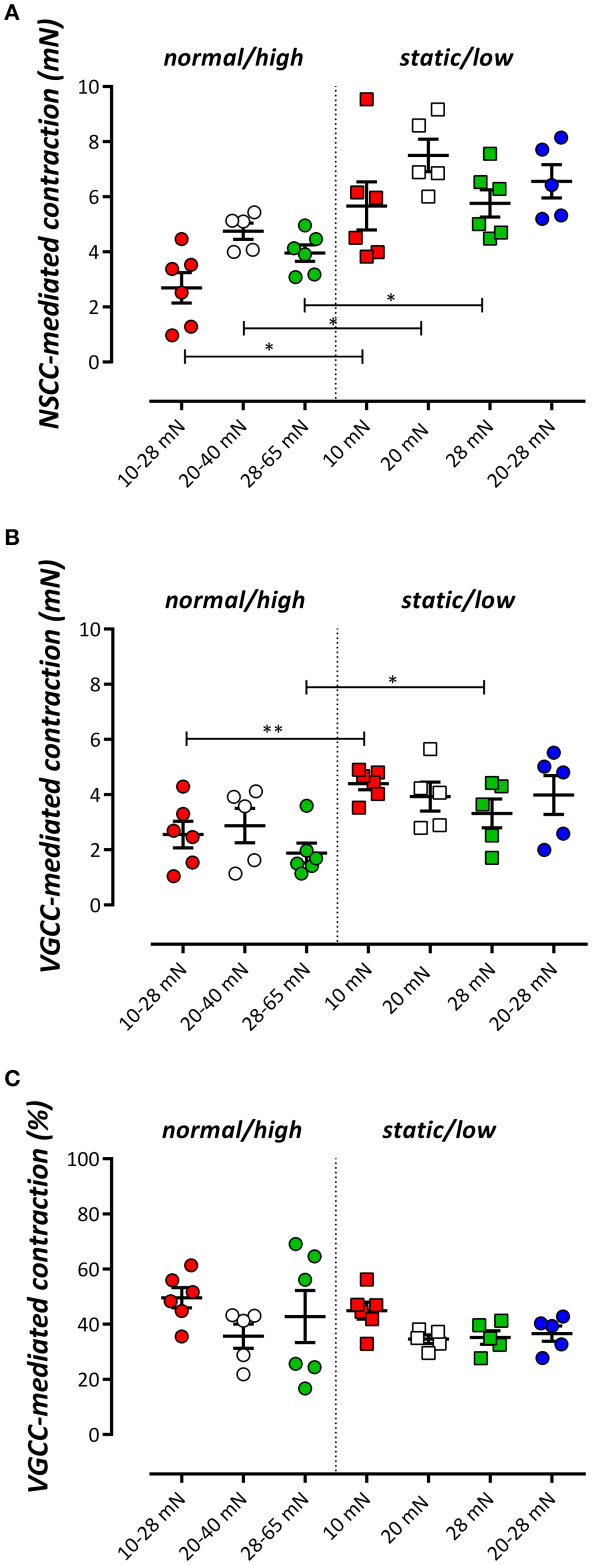
The tonic contraction by 2 μM PE mediated by NSCC Ca^2+^ influx **(A)** or VGCC Ca^2+^ influx **(B)** in the presence of 300 μM L-NAME. VGCC were inhibited with 35 μM diltiazem. The relative amount of VGCC-mediated contraction to the total contraction by PE is shown in **(C)**. ^*^P < 0.01, ^**^P < 0.05 cyclic vs. corresponding static stretch. *N* = 5–6, one-way ANOVA with Tukey's multiple comparison test.

After 600 s, the PE-induced tonic contraction was inhibited with 35 μM diltiazem, causing partial relaxation of the contraction by PE (Figures 3D,E). The contraction remaining after addition of diltiazem is due to Ca^2+^ influx via NSCC (Fransen et al., 2015). Figure 5 shows the contraction due to VGCC (Figure 5A) and NSCC (Figure 5B) Ca^2+^ influx in the different conditions. VGCC-mediated contractions after normal to high cyclic stretch conditioning were significantly smaller than these after static to low cyclic stretch conditioning. For NSCC-mediated contractions, all contractions after cyclic stretch (except the low amplitude 20–28 mN condition) were significantly lower than the contractions measured after static stretch. Relatively, diltiazem inhibited about 40% of the contraction in all conditions studied, whereas 60% of the tonic contraction was due to NSCC Ca^2+^ influx (Figure 5C). There were no differences between the relative amount of VGCC-mediated contraction between the different cyclic stretch conditions.

Because cyclic stretch has been described to activate complex mechanotransduction networks, in which there might be an important contribution of Rho-kinase signaling (Yang et al., 2014), we tested whether Rho kinase inhibition with Y-27632 (3 μM) was responsible for some of the differences between low (20–28 mN) and physiological (20–40 mN) amplitude cyclic stretch conditioning (Supplementary Figure 1). Y-27632 inhibited both phasic and tonic contractions by 2 μM PE, but the inhibition was independent of the amplitude of cyclic stretch. Also, the VGCC mediated contraction (as measured by inhibition of VGCC with 35 μM diltiazem) was reduced with Y-27632 and independent of the stretch amplitude.

### Static vs. cyclic stretch: focus on EC and NO

**Figure 6:**
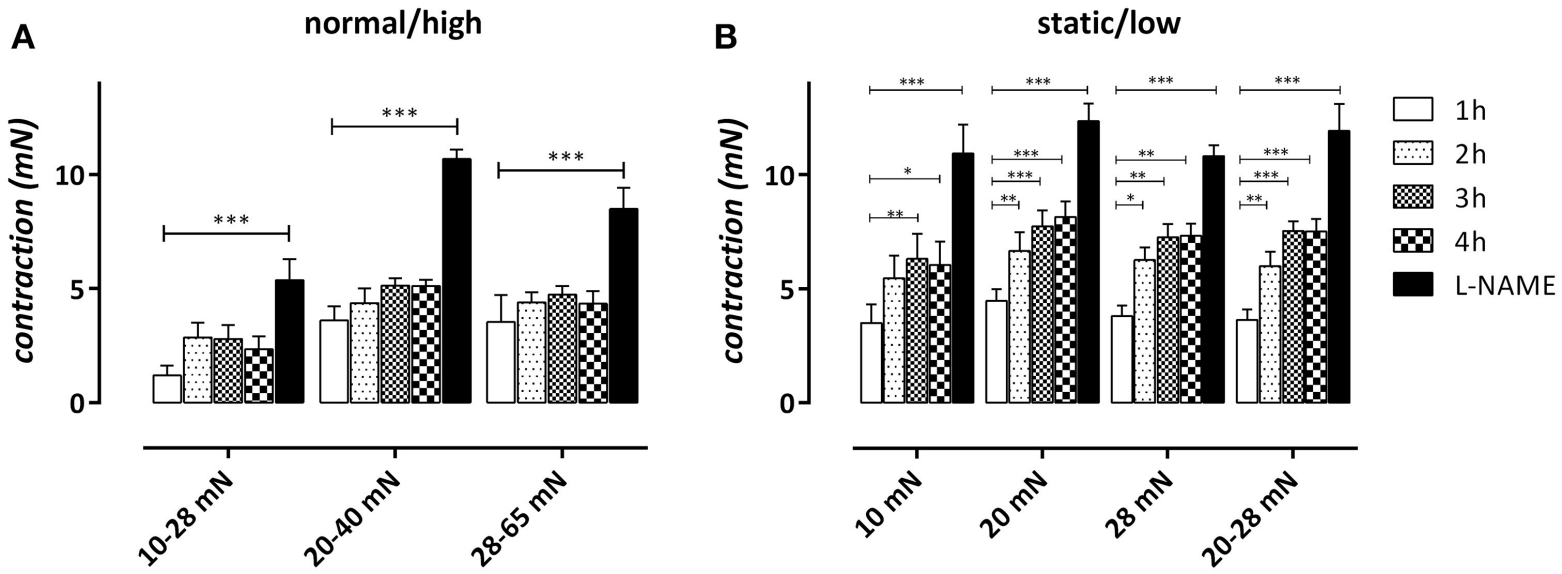
“Steady-state” effects of 2 μM PE in aortic segments subjected to normal/high cyclic stretch **(A)** or static/low cyclic stretch **(B)**. Contractions were measured 1, 2, 3, and 4 h after mounting and after 300 μM L-NAME was added to determine the maximal contraction by inhibiting relaxing basal NO release. ^*^P < 0.05, ^**^P < 0.01, ^***^P < 0.001 vs. 1 h. *n* = 5–6, one-way ANOVA with Dunnett's multiple comparison test.

We have shown before that in basal conditions, non-stimulated release of NO in mouse aortic segments spontaneously decreases with time after mounting the aortic segments in the organ bath in static, isometric conditions (van Langen et al., 2012). To test whether cyclic stretch at different amplitudes affects this spontaneous decrease of basal NO efficacy, aortic segments were subjected to cyclic stretch at 10 Hz at the different static and cyclic stretch conditions and contracted every hour with 1 μM PE. After 600 s, the amplitude of the contraction was measured and plotted as a function of time after mounting (Figure 6). After 4 h, eNOS was inhibited with 300 μM L-NAME to determine the maximal effect of 2 μM PE in the absence of basal NO release.

In static stretch conditions (10, 20, and 28 mN) the contraction by PE increased with time after mounting and the increase was significant after 2 or 3 h (Figure 6B). In the cyclic stretch conditions at 20–40, 10–28, and 28–65 mN, the time-dependent increase of contraction was non-significant up to 4 h after mounting (Figure 6A). For the low amplitude stretch at 20–28 mN, however, the time-dependent increase was already significant 2 h after mounting and was similar to the time-dependent increase of contraction at the static stretch of 20 mN. Inhibition of eNOS with 300 μM L-NAME after 4 h increased the contraction in all conditions.

**Figure 7:**
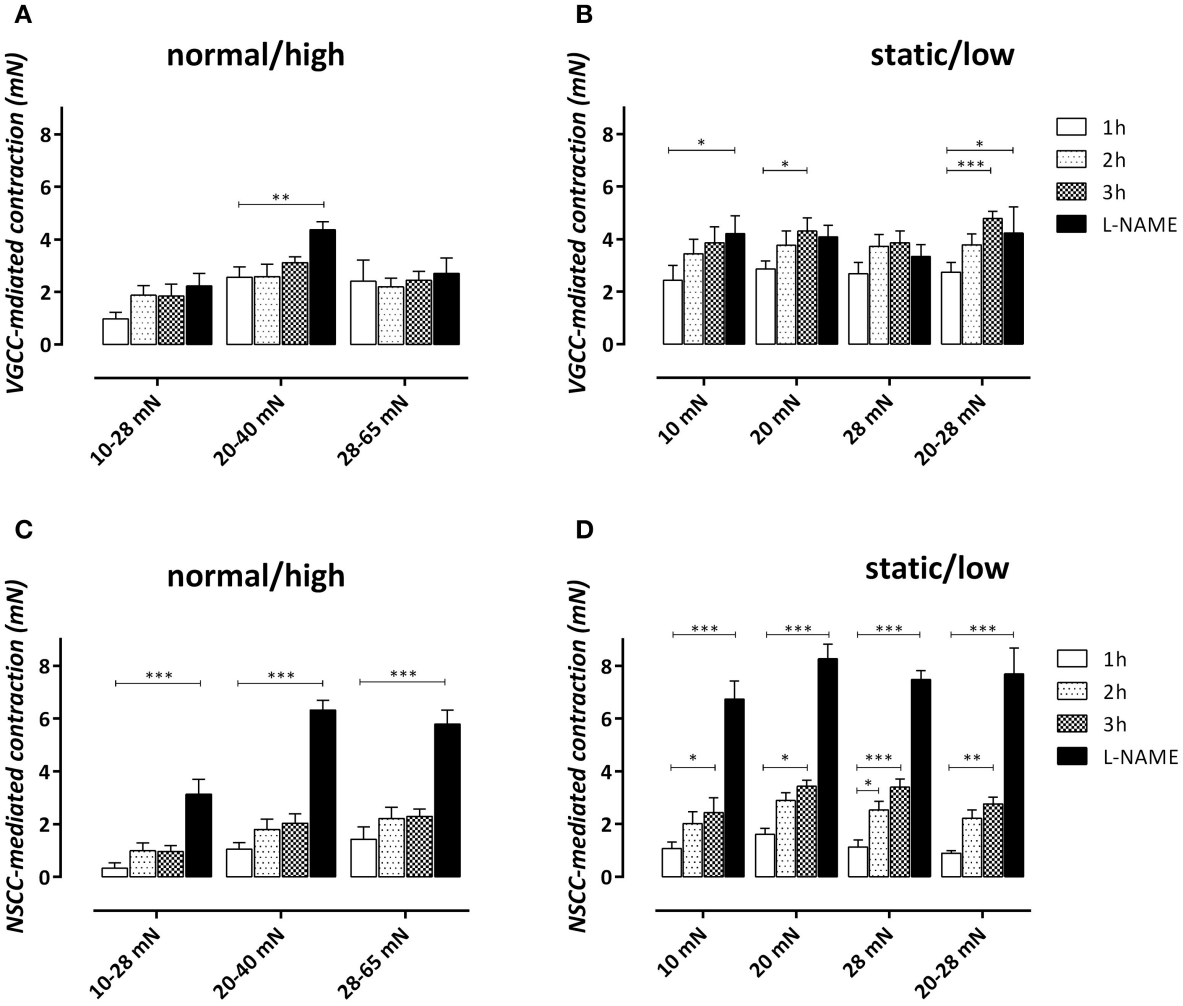
VGCC **(A,B)**- and NSCC **(C,D)**-mediated contractions by 2 μM PE in aortic segments subjected to cyclic stretch of normal/high amplitude **(A,C)** or static/low cyclic stretch **(B,D)** determined after addition of 35 μM diltiazem at 1, 2, and 3 h after mounting. After 4 h, 300 μM L-NAME was added to determine the maximal VGCC- or NSCC-mediated contraction by inhibiting relaxing NO release. ^*^P < 0.05, ^**^P < 0.01, ^***^P < 0.001 vs. 1 h. *n* = 5–6, one-way ANOVA with Dunnett's multiple comparison test.

The PE-induced contractions at 1, 2, and 3 h were inhibited with 35 μM diltiazem to reveal the contribution of VGCC Ca^2+^ influx to the total contraction (Figures 7A,B). The contraction remaining after inhibition of VGCC-mediated contraction with diltiazem is due to Ca^2+^ influx via NSCC (Figures 7C,D).

In the normal/high cyclic stretch conditions, the VGCC-mediated contraction did not significantly increase with time after mounting. Only in the 20–40 mN stretch condition, L-NAME caused a significant increase of the VGCC-mediated contraction, suggesting that “removal” of basal NO release only affected VGCC-mediated contractions in “normal” stretch conditions. In static/low cyclic stretch conditions, the contraction displayed a small, but significant increase of VGCC-mediated contraction at 3 h (20 and 20–28 mN) or after addition of L-NAME (10 mN) (see Supplementary Figure 3). The NSCC-mediated contraction, which was smaller than the VGCC-mediated contraction at 1 h in all conditions, increased with time after mounting in all static/low cyclic stretch conditions. There was no significant time-dependency observed in the normal/high cyclic stretch conditions. In all conditions, inhibition of eNOS with L-NAME, and hence, inhibition of basal NO release, caused a highly significant increase of NSCC-mediated contraction, suggesting that the NSCC-mediated contraction is very dependent on the basal release of NO. This observation also suggests that the increase of NSCC-mediated Ca^2+^ influx by removal of basal NO production does not cause an increase of the VGCC-mediated contraction, indicating that both types of contractions are differently affected by NO and are not interrelated.

### Relaxation of stretched segments with acetylcholine

**Figure 8:**
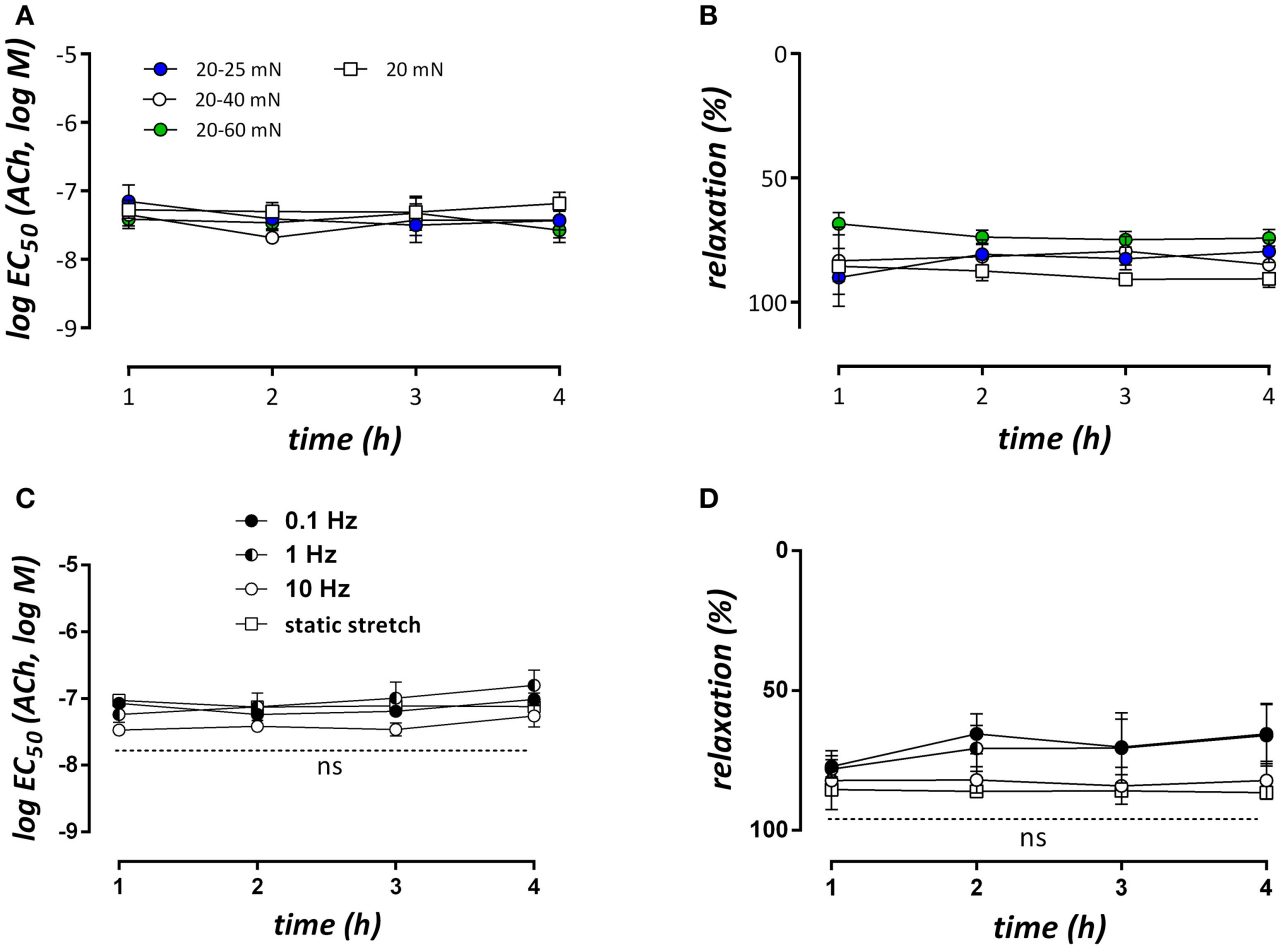
LogEC_50_- and E_max_-values for ACh-mediated relaxations of contractions induced by 2 μM PE at different time intervals after mounting the segments (*n* = 5, each). In **(A,B)** segments were conditioned at 10 Hz and different stretch amplitudes (no cyclic stretch, 20–25, 20–40, and 20–60 mN), whereas in **(C,D)** the stretch amplitude was 20 mN (20–40 mN), but the frequency was 10, 1, or 0.1 Hz. All contractions and relaxations were measured in isometric conditions at 20 mN. *n* = 5–6, two-way ANOVA with Tukey's multiple comparison test.

Segments conditioned at different amplitudes or frequencies of cyclic stretch were contracted with 2 μM PE in isometric conditions and then relaxed by stimulating NO release from the endothelial cells with increasing concentrations of ACh at 1, 2, 3, or 4 h after mounting. Increasing the cyclic stretch amplitude from 0 (static 20 mN) to 5 (20–25 mN), 20 (20–40 mN), or 40 (20–60 mN) mN had no significant effect at any time interval on the log EC_50_ of ACh (Figure 8A) or the maximal relaxation (Figure 8B). Similar results were obtained for the different cyclic stretch frequencies (Figures 8C,D).

### Frequency of cyclic stretch

**Figure 9:**
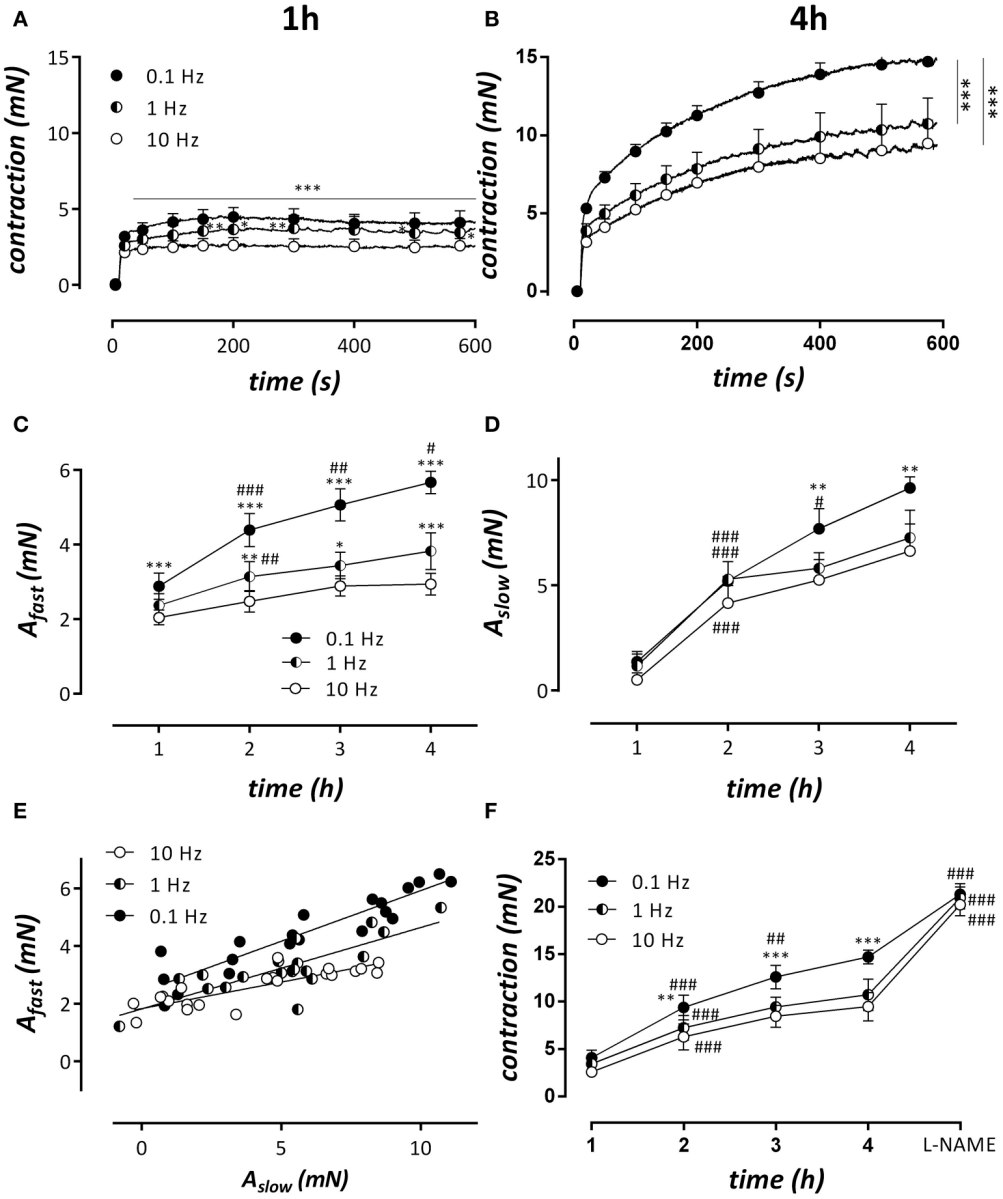
Cyclic stretch between 80 and 120 mm Hg was applied for 1, 2, 3, and 4 h at stretch frequencies of 0.1, 1, and 10 Hz after which the isometric contractions by 2 μM PE were measured at 80 mm Hg. After the isometric contraction at 4 h, 300 μM L-NAME was added to block basal NO release. **(A,B)** Show isometric contractions by PE at the different frequencies at 1 h **(A)** and 4 h **(B)** after mounting. Traces at 1, 2, 3, and 4 h were analyzed with a double exponential function revealing amplitudes of fast (A_fast_, **C**) and slow (A_slow_) force components as a function of time after mounting. **(E)** Shows the relationship between fast and slow component at the different frequencies. All slopes were significantly different from zero and significantly different from each other (*p* < 0.001, pearson correlation). In **(F)**, the “steady-state” contraction after 600s in the different conditions is shown. ^*^P < 0.05, ^**^P < 0.01, ^***^P < 0.001 vs. 10 Hz in **(A–F)**; ^#^*P* < 0.05, ^*##*^*P* < 0.01, ^*###*^*P* < 0.001 vs. previous time point. *n* = 5–6, two-way ANOVA with Tukey's multiple comparison test.

In the following experiments aortic segments were subjected to physiological stretch between 20 and 40 mN at different frequencies: 10, 5, 2.5, 1.25, 1, and 0.1 Hz. This stretch was interrupted every hour after mounting to measure isometric contractions by 2 μM PE (Figure 9). Because we found no differences in contractility after cyclic stretch periods with frequencies of 10, 5, 2.5, and 1.25 Hz, only the conditions at 10, 1, and 0.1 Hz are shown. In all stretch conditions, the effects of PE increased with time but more in the low frequency than in the high stretch settings (Figures 9A,B,F). After the measurements at 4 h, 300 μM L-NAME was added to measure the amount of relaxing effect induced by basal release of NO (Figure 9F). In all frequency conditions, the contraction in the presence of L-NAME was equal, suggesting that the amount of basal NO release differed between the frequency stretch conditions at 2, 3, and 4 h after mounting. Contractions at 1, 2, 3, and 4 h after mounting were analyzed with a double exponential function to reveal the fast and slow components of contraction. Previously, we have shown that these components correspond to the phasic and tonic contraction of PE (Fransen et al., 2015). Figure 9C shows that the fast component is very dependent on the frequency of cyclic stretch. A_fast_ increased more over time at 0.1 than at 1 or 10 Hz, and was significantly increased at 0.1 or 1 Hz at all-time intervals after mounting compared to the physiological frequency of 10 Hz. For the slow component or phasic contraction, the time-dependent increase was also evident and more pronounced at 0.1 Hz. At 3 and 4 h after mounting, A_slow_ was significantly increased at this frequency when compared with 10 Hz. When A_fast_ was plotted against A_slow_ (Figure 9E), there was a correlation between both parameters in all conditions, but the slope of the linear relationship was significantly (*P* < 0.001) higher at 0.1 and 1 Hz than at 10 Hz (respectively 0.35, 0.28, and 0.18).

## Discussion

The present study demonstrated that contractile properties of mouse aortic segments depend on the amplitude and frequency of cyclic stretch. When compared with static stretch or low amplitude (<10 mN) or low frequency (0.1 Hz) cyclic stretch, cyclic stretch at physiological amplitudes (>10 mN) and frequencies (1–10 Hz), caused (1) a decrease of baseline contraction, which is probably due to attenuated baseline Ca^2+^ influx and which leads to a lower contractile Ca^2+^ content of the VSMC SR; (2) lower tonic contractions when segments are contracted with the α_1_ adrenoceptor agonist, PE; (3) a non-significant or mild increase of contractions by PE with time after mounting, suggestive for better conservation of basal NO release, and (4) unaltered relaxation of PE-precontracted segments by addition of ACh to stimulate NO release.

Each heartbeat (100,000 heartbeats per day for humans up to 800,000 beats per day for mice) imposes a pulsatile pressure in the aorta that generates EC and VSMC stretch (normally ± 10%) in the vessel wall. The present study investigated the consequences of lower (4%) and higher (14%) than normal (12%) cyclic stretch. Cyclic stretch together with shear stress represent the most important mechanical stimuli in elastic arteries, but both physical parameters have differential effects on both EC (Peng et al., 2003) and VSMC function (Thacher et al., 2009). Although, the effects of shear stress, flow and pressure are extensively studied especially in the smaller blood vessels, the effects of cyclic stretch in aortic segments *ex-vivo* are underexposed, and almost exclusively studied in cultured cells grown on elastomer-bottomed culture plates, mostly at very low stretch amplitudes (2–5%) (Anwar et al., 2012; Mantella et al., 2015). These *in vitro* studies have not led to coherent conclusions, and lack the cross-talk between EC and VSMC, and their extracellular matrix (ECM), which is of important physiological relevance (Mantella et al., 2015). It has been shown that both amplitude and frequency of cyclic stretch applied to adult human vascular smooth muscle cells cultured within three dimensional tubular collagen gel constructs determine the phenotype of the smooth muscle cells, elastin mRNA expression, and the orientation of the smooth muscle cells within the tubular structure (Venkataraman et al., 2014). After 48 h of stretching (10%, 1 Hz), VSMCs of aorta were less rigid compared with controls (Dinardo et al., 2014). Also the ECM may be involved in cyclic stretch-induced phenotypic modulation: while the application of static or slowly varying mechanical forces accelerates the digestion-induced breakdown of ECM, an elevated cyclic stretch frequency can have a protective role (Jesudason et al., 2007). Therefore, continued progress in this field of research is hampered by technical limitations in studying the effect of cyclic stretch *in vivo*, or *ex vivo* on intact arterial segments. The present study uses the ROTSAC, an in-house developed *ex vivo* platform, which has been used to measure isobaric aortic segment compliance and stiffness parameters (Leloup et al., 2016). The platform allows for the application of cyclic stretch protocols to isolated aortic segments at preloads, which can be roughly correlated with (patho)physiological pressures (see Table 1) and frequencies, in conditions with an intact cross-talk between EC, VSMC, and the ECM. To allow comparisons with classical organ bath experiments, in which vaso-reactivity is always measured in isometric conditions, all measurements in the ROTSAC were also repeated in isometric conditions by applying an afterload to the diastolic preload after a stretch protocol (see Figure 1).

### Phasic contractions by α_1_-adrenoceptor stimulation with PE

Acute stretch of mouse thoracic aorta elicits two types of intracellular calcium responses in the VSMC. One was a phasic calcium discharge generated by the sarcoplasmic reticulum (SR), the second was a tonic response produced by the activation of stretch-sensitive non-selective cationic channels (NSCC) allowing external Ca^2+^ entry (Fanchaouy et al., 2007). Previously, we have shown that high static stretch of aortic segments increases baseline Ca^2+^ influx in VSMC, mainly by depolarizing the VSMC and activating baseline VGCC Ca^2+^ influx and contraction. Concomitantly, there was a decrease of the contractile Ca^2+^ content of the SR. This suggests that static stretch causes emptying of the contractile SR Ca^2+^ store (Fanchaouy et al., 2007; De Moudt et al., 2017). Aortic segments subjected to cyclic stretch (amplitude > 10 mN) also displayed lower PE-induced phasic contractions, even at preloads which are accompanied with a “large” SR contractile Ca^2+^ content in static conditions. Similarly, the fast component of contraction, referring to the phasic contraction in the absence of extracellular Ca^2+^, was significantly smaller for physiological cyclic stretch at 20–40 mN at 1 or 10 Hz. These results suggest that the lower contractile SR Ca^2+^ content in these high frequency stretched segments is related with a lower cytoplasmic Ca^2+^ influx in baseline conditions (Leloup et al., 2015a) when compared with segments subjected to static stretch, cyclic stretch with low (<10 mN) amplitudes, or cyclic stretch at very low frequencies. Indeed, removal of external Ca^2+^ caused a larger decrease of baseline force in static segments than in segments which were cyclically stretched at sufficient amplitude. This lower resting tension, which may correlate with a low cytoplasmic Ca^2+^ content of the VSMC may have prominent effects on the VSMC phenotype since cyclic stretch seems to be essential in maintaining the highly differentiated VSMC contractile phenotype (Gilbert et al., 2014) and has been further been shown to be highly contingent on VSMC calcium handling (Berra-Romani et al., 2008; Matchkov et al., 2012). Calcium, which is a known second messenger in excitation-contraction coupling, can regulate VSMC selective gene expression programs, a new paradigm coined excitation-transcription coupling (Wamhoff et al., 2006). In this system, several calcium-dependent mediators, such as RhoA/ROCK, calcineurin, and calmodulin kinase IV (CaMKIV), regulate the activity and localization of transcription factors that are essential in the maintenance of the contractile phenotype. Exposure to pathological cyclic stretch (low amplitude of ≤5%) increases intracellular calcium levels, which may activate gene expression programs that induce a phenotypic switch to synthetic VSMC. This synthetic phenotype is characterized by an increased proliferation rate, increased secretion of matrix metalloproteinases (MMP and increased production of ECM proteins such as type-1 collagen (Anwar et al., 2012). It has been described that in rat aortic vascular smooth muscle cells, seeded on type I collagen-coated flexible silicone bottom plates, cyclic stretch (10%, 1.25 Hz) repressed the expression of ROCK1 (Yang et al., 2014). In the present experiments, Rho kinase inhibition with Y-27632 did, however, not differentially affect isometric contractions of mouse aortic segments conditioned at low (20–28 mN) or physiological (20–40 mN) cyclic stretch, suggesting a minor role of Rho kinase in affecting stretch-dependency at these preloads. However, we cannot exclude larger effects of Rho kinase inhibition at higher amplitudes of cyclic stretch or higher preloads. Coincidentally or not, all the above mentioned phenomena are related with the development of arterial stiffening, suggesting that the basal increase of intracellular Ca^2+^ in VSMC may be one of the first events leading to arterial stiffness (Anea et al., 2010; Janić et al., 2014). Whether extended periods of cyclic stretch of variable amplitude or frequency can induce arterial stiffness combined with phenotypic switch of the VSMC in the cyclic stretch conditions discussed in these experiments, will need further investigation.

### Tonic contractions by α_1_-adrenoceptor stimulation with PE

After the phasic contraction, re-addition of extracellular Ca^2+^ to the 0Ca solution containing 2 μM PE, caused tonic contractions which were significantly smaller in segments subjected to high frequency (10 Hz) or normal/high amplitude cyclic stretch, compared to segments subjected to static or low amplitude cyclic stretch or cyclic stretch with low frequency (0.1, 1 Hz) (Figures 3, 9). In the segments that were maintained static or stretched at low amplitude, the tonic contraction was linearly related to the phasic contraction (Figure 4), whereas this relationship was absent for the segments stretched at high amplitude. Similar observations were made when the fast component of contraction (A_fast_, a measure of the phasic contraction) was plotted against the slow component of contraction (A_slow_, a measure of the tonic contraction) at clamp frequencies of 0.1, 1, and 10 Hz. Although the slope at 10 Hz was significantly different from zero, the slope was rather flat and significantly increased at 1 and further at 0.1 Hz. This indicates that at low frequency but normal amplitude of cyclic stretch, a higher phasic contraction is followed by a higher tonic contraction. Which stretch-dependent transduction system is sensing the amplitude and frequency of the stretch and thereby couples or uncouples SR Ca^2+^ release and subsequent Ca^2+^ influx is a topic for further investigation.

In the presence of L-NAME, hence the absence of basal NO release, the tonic contraction by PE was mainly caused by NSCC-mediated Ca^2+^ influx (60%). Cyclic stretch (7–18% stretch, inter stimulus interval of 6 s with 3 s on and 3 s off) has been described to decrease TRPC4 protein levels in isolated rat vascular smooth muscle cells, which was related to reduced capacitive calcium entry in WKY but not in SHR (Lindsey et al., 2008; Lindsey and Songu-Mize, 2010). In general, hypertension seems to have differential effects on TRPC channels, but how these channels react to cyclic stretch has not been studied in mouse aorta (Dietrich et al., 2006; Liu et al., 2009; Noorani et al., 2011). Previously, we have shown that an increase in static stretch preload causes a higher contribution of NSCC-mediated contractions (De Moudt et al., 2017). Nevertheless, both the VGCC- and NSCC-mediated contractions were significantly lower in cyclic stretch conditions (except for the low amplitude cyclic stretch) when compared with the static stretch conditions, suggesting that cyclic stretch reduces the Ca^2+^ load of the VSMC even at high preloads (28–65 mN).

### Role of NO release during cyclic stretch

Basal release of NO in non-stimulated mouse aortic segments spontaneously decreases with time after mounting the aortic segments in the organ bath in static, isometric conditions (van Langen et al., 2012). Elastic arteries are constantly exposed to high levels of cyclic stretch and display remarkably higher basal release of endothelial NO compared to muscular arteries (Leloup et al., 2015b). Therefore, we hypothesized that cyclic stretch may play a major role in regulating basal release of NO in aortic segments, since, aside from shear stress, it also represents an important mechanical parameter that is not commonly simulated in an isometric *ex vivo* environment. At least, this mechanical parameter has been shown to be essential for the normal development, homeostasis and remodeling of the vasculature (Chatterjee et al., 2015; Thorin-Trescases and Thorin, 2016). Similar to observations in VSMC (Fanchaouy et al., 2007; Lindsey and Songu-Mize, 2010), exposure of cultured endothelial cells to cyclic strain (10%, 1 Hz) induces elevations of cytosolic Ca^2+^ through mobilization of intra and extracellular calcium pools (Rosales et al., 1997). Furthermore, cyclic stretch has been described to regulate eNOS synthesis and activity, which depend on (i) calcium influx through stretch-sensitive ion channels, and (ii) eNOS phosphorylation by phosphoinositide 3-kinase (PI3K)/protein kinase B (Akt) signaling pathways (Peng et al., 2003; Takeda et al., 2006), highlighting a role for cyclic stretch in basal NO release.

The present study showed that endothelial NO efficacy in basal, non-stimulated conditions is highly contingent on cyclic stretch stimulation, and is dependent on both stretch-frequency and stretch-amplitude. The time-dependent increase of PE-induced contractions as a measure of the contraction-depressant effect of basal NO release was attenuated in cyclic stretch conditions with physiological amplitudes (10–28, 20–40, and 28–65 mN) compared with static or low amplitude cyclic stretch (Figure 6). The contraction-depressant effect of basal NO was evident for the contractions mediated by VGCC, but especially for the contractions via NSCC, confirming that NSCC-mediated Ca^2+^ influx is more NO-sensitive than VGCC-mediated Ca^2+^ influx (De Moudt et al., 2017). Different cyclic stretch frequencies (at 25–40 mN) further revealed that time-dependent loss of basal NO efficacy *ex vivo* was significantly attenuated at 1 or 10 Hz, compared to a 0.1 Hz frequency. The largest effects of cyclic stretch frequency were observed on the fast component of the PE-elicited contraction, which was extremely time-dependent at 0.1 Hz. It seemed that in all experimental conditions, only those conditions featuring both a sufficient cyclic stretch amplitude (>10 mN or extrapolated 20 mm Hg) and frequency (>1 Hz) were able to promote the maintenance of basal NO release or efficacy. In none of the conditions, stimulated NO release by ACh was affected, suggestive for the conservation of an intact endothelial monolayer at low or even very high amplitudes or frequencies (see Supplementary Figure 2).

It has been described that pathophysiological levels of cyclic stretch result in a reduction in endothelial NO production, the hallmark of endothelial dysfunction. As such, pathological cyclic stretch induces an EC phenotype with decreased vasodilatory capacity. Since reduced NO bioavailability has been shown to increase arterial stiffness in rodents (Isabelle et al., 2012; Leloup et al., 2014), whereas enhanced NO bioavailability can improve large artery compliance in humans (Bellien et al., 2010) and animal models (Fitch et al., 2006; Sindler et al., 2011), improving NO bioavailability by targeting cyclic stretch-dependent EC phenotypic modulation might also serve as a novel therapeutic strategy. The endothelial phenotypic switch in the absence of physiological cyclic stretching causes the VSMC tone to increase, leading to the development of arterial stiffness, and predicting future progression to hypertension and vascular end-organ damage. Subsequent VSMC phenotype switch will promote further arterial stiffening, and hence, also the VSMC phenotype switch might represent an interesting therapeutic target.

In summary, cyclic stretch causes significant alterations of the isometric contractions produced by α_1_-adrenoceptor stimulation of mouse aortic segments with PE and the role of basal NO release herein. Both cyclic stretch amplitude and frequency contribute in setting the mechanical properties of isolated aortic segments. *In vivo* hemodynamic alterations caused by hypertension and arterial stiffness result in chronic exposure to pathological cyclic stretch, leading to phenotypic modulation of EC and VSCM and resultant arterial remodeling (Thorin-Trescases and Thorin, 2016). As such, mechanotransduction of pathological cyclic stretch may underlie the amplificatory relationship between hypertension and arterial stiffness in elastic arteries.

## Author contributions

AL, SD, CV, and PF designed, performed and analyzed the experiments. AL and PF drafted the work, whereas all authors approved a final version and agree to be accountable for all aspects of the work in ensuring that questions related to the accuracy or integrity of any part of the work are appropriately investigated and resolved.

### Conflict of interest statement

The authors declare that the research was conducted in the absence of any commercial or financial relationships that could be construed as a potential conflict of interest. The reviewer JVT and handling Editor declared their shared affiliation.

## Supplementary material

Supplementary Figure 1 Tonic and phasic contractions by 2 μM PE were studied in the absence and presence of 3 μM of the Rho-kinase inhibitor Y-27632. In **(A,B)**, phasic contractions are shown after conditioning at 20–40 mN (black) and 20–28 mN (red), two conditions in which we observed significant differences between the isometric contractions. Y-27632 caused inhibition of the phasic contraction (**B** vs. **A**), which is summarized for the area under the curve (AUC) in **(C)**. From **(D)**, it is clear that Y27632 inhibited the phasic contraction by about 50% as well after conditioning at 20–40 as 20–28 mN. Hence the difference between the phasic contractions between the two conditions is not due to calcium sensitization differing between low and normal cyclic stretch. We also determined the PE-induced tonic contraction after re-addition of extracellular calcium **(E,F)**. The tonic contraction in the presence of 3 μM Y27632 was reduced after conditioning with cyclic stretch of 20–28 and 20–40 mN and the difference between the 20–28 and 20–40 mN tonic contraction persisted after Rho kinase inhibition. The contraction inhibited with 35 μM diltiazem (VGCC mediated contraction) was also significantly reduced after Rho kinase inhibition. Although we could not demonstrate significant differences between the 20–28 and 20–40 mN conditions for VGCC mediated tonic contractions before and after Rho kinase inhibition, similar trends as observed for the tonic contractions were evident. These results suggested that a role of Rho kinase in the establishment of the different contraction at different cyclic stretch amplitudes could not completely ruled out. It is at least clear that the difference in phasic and tonic contraction between 20–28 and 20–40 mN is for the largest part due to altered calcium mobilization (phasic contraction) and calcium influx (tonic contraction) and for a minor part to calcium sensitivity. *n* = 5, **(A,B)**, two way ANOVA with Sidak's multiple comparison; **(C,E,F)** one way ANOVA with Tukey's multiple comparison test; **(D)**, paired *t*-test.

Supplementary Figure 2 Reversibility of stretch pre-conditioning. IP_3_-mediated phasic contractions by 2 μM PE in the absence of external calcium were elicited in different conditions (all in the presence of 3 μM Y-27632). IP_3_-mediated phasic contractions by PE were typically larger at the low amplitude cyclic stretch (20–28 mN) condition than at the higher cyclic stretch (20–40 mN, 28–65 mN) conditions (**A,C**). When segments were conditioned for 30 min with stretch of higher amplitude (red, 20–40 or 28–65 mN, **A,C**) the IP3 mediated phasic contraction after conditioning at 20–28 mN increased (red, **B,D**) and vice versa (black curves). The number of experiments is 2 for **(A,B)** and 1 for **(C,D)**.

Supplementary Figure 3 In 6 mice we depolarized the segments, which were subjected to conditioning cyclic stretch of 20–28, 20–40, 10–28, and 28–65 mN, and measured the isometric contraction induced by 50 mM external K^+^. We compared this contraction with the isometric contraction by 2 μM PE in the absence and presence of 35 μM diltiazem to reveal the relative contribution of VGCC to the PE-induced contraction. From the figure it is clear that the VGCC-mediated contraction in the different conditions (triangles, right Y-axis) was dependent on the conditioning cyclic stretch in exactly the same way as the contraction mediated by depolarization with 50 mM K^+^ (squares, left y-axis). Contractions were somewhat larger than the contractions shown in the submitted manuscript, because the mice were older (8 months), but the overall data were qualitatively the same. *n* = 6, one way ANOVA with Tukey's multiple comparison test.
