# Supplementary figures and images for: Cyclic Stretch Alters Vascular Reactivity of Mouse Aortic Segments
Source: Front Physiol. 2017 Oct 30;8:858. doi: 10.3389/fphys.2017.00858 (PMC5674939; doi:10.3389/fphys.2017.00858)

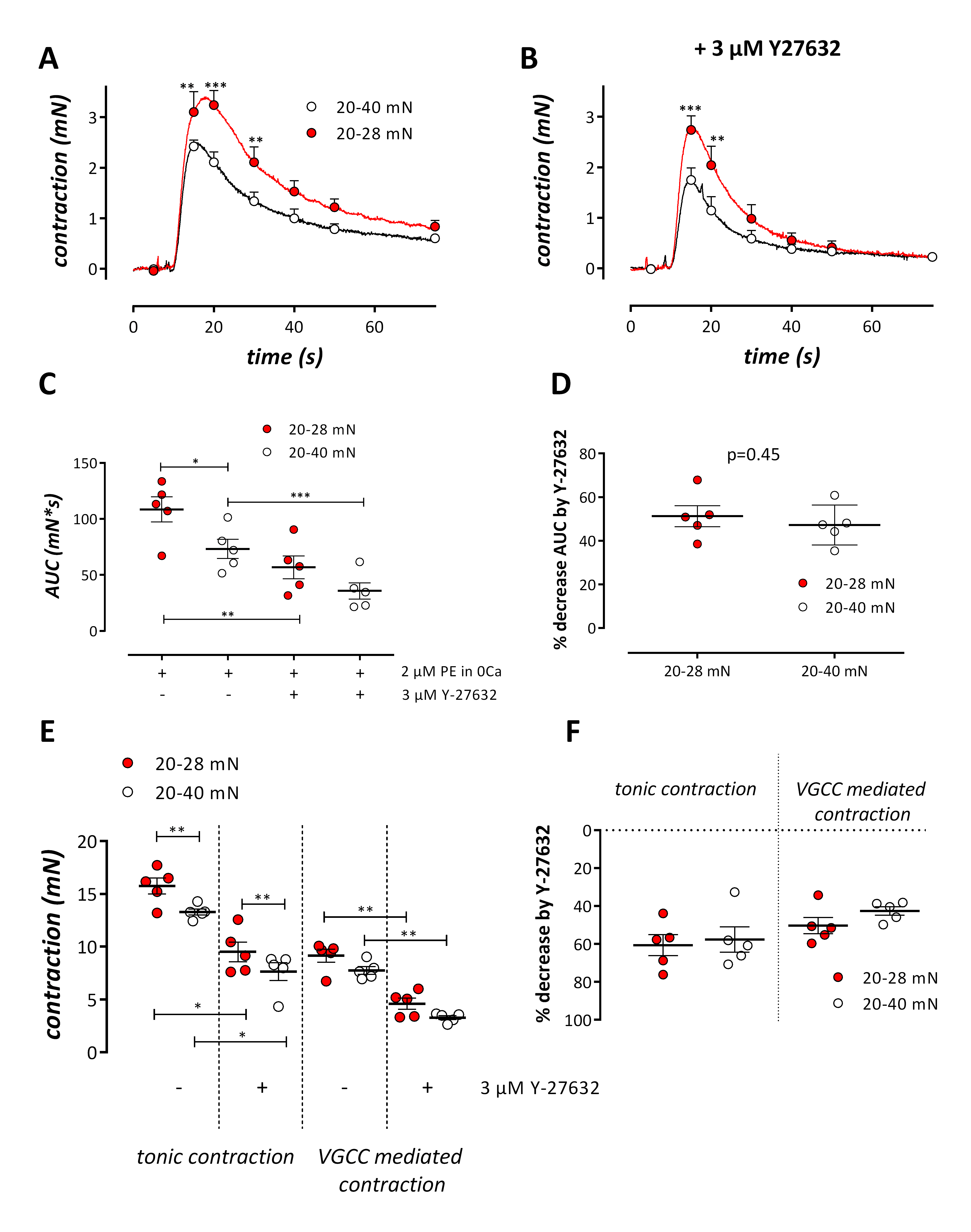

Supplement: Supplementary Figure 1 — Tonic and phasic contractions by 2 μM PE were studied in the absence and presence of 3 μM of the Rho-kinase inhibitor Y-27632. In (A,B), phasic contractions are shown after conditioning at 20–40 mN (black) and 20–28 mN (red), two conditions in which we observed significant differences between the isometric contractions. Y-27632 caused inhibition of the phasic contraction (B vs. A), which is summarized for the area under the curve (AUC) in (C). From (D), it is clear that Y27632 inhibited the phasic contraction by about 50% as well after conditioning at 20–40 as 20–28 mN. Hence the difference between the phasic contractions between the two conditions is not due to calcium sensitization differing between low and normal cyclic stretch. We also determined the PE-induced tonic contraction after re-addition of extracellular calcium (E,F). The tonic contraction in the presence of 3 μM Y27632 was reduced after conditioning with cyclic stretch of 20–28 and 20–40 mN and the difference between the 20–28 and 20–40 mN tonic contraction persisted after Rho kinase inhibition. The contraction inhibited with 35 μM diltiazem (VGCC mediated contraction) was also significantly reduced after Rho kinase inhibition. Although we could not demonstrate significant differences between the 20–28 and 20–40 mN conditions for VGCC mediated tonic contractions before and after Rho kinase inhibition, similar trends as observed for the tonic contractions were evident. These results suggested that a role of Rho kinase in the establishment of the different contraction at different cyclic stretch amplitudes could not completely ruled out. It is at least clear that the difference in phasic and tonic contraction between 20–28 and 20–40 mN is for the largest part due to altered calcium mobilization (phasic contraction) and calcium influx (tonic contraction) and for a minor part to calcium sensitivity. n = 5, (A,B), two way ANOVA with Sidak's multiple comparison; (C,E,F) one way ANOVA with Tukey's multiple c [file Image1.JPEG]

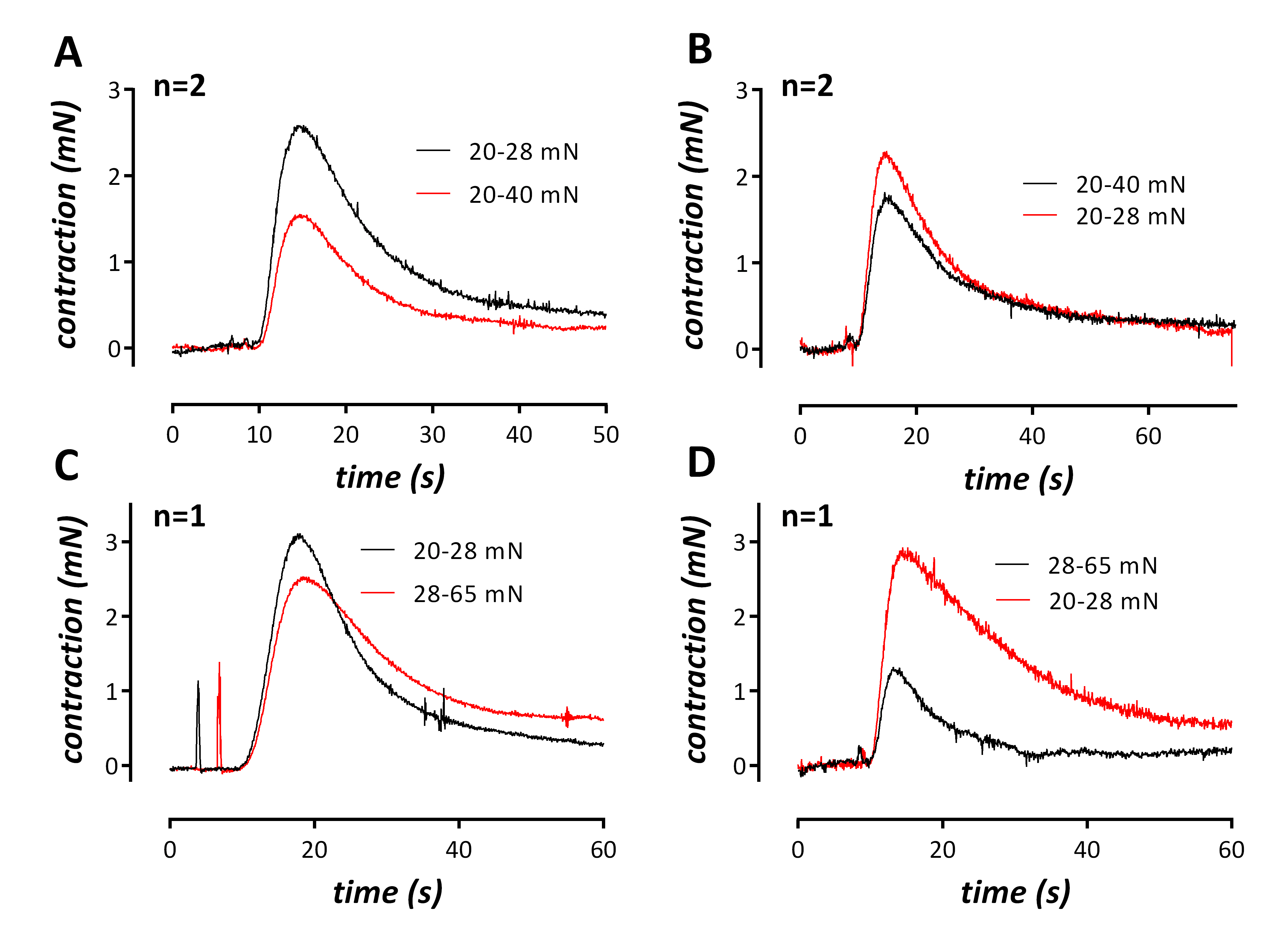

Supplement: Supplementary Figure 2 — Reversibility of stretch pre-conditioning. IP3-mediated phasic contractions by 2 μM PE in the absence of external calcium were elicited in different conditions (all in the presence of 3 μM Y-27632). IP3-mediated phasic contractions by PE were typically larger at the low amplitude cyclic stretch (20–28 mN) condition than at the higher cyclic stretch (20–40 mN, 28–65 mN) conditions (A,C). When segments were conditioned for 30 min with stretch of higher amplitude (red, 20–40 or 28–65 mN, A,C) the IP3 mediated phasic contraction after conditioning at 20–28 mN increased (red, B,D) and vice versa (black curves). The number of experiments is 2 for (A,B) and 1 for (C,D). [file Image2.JPEG]

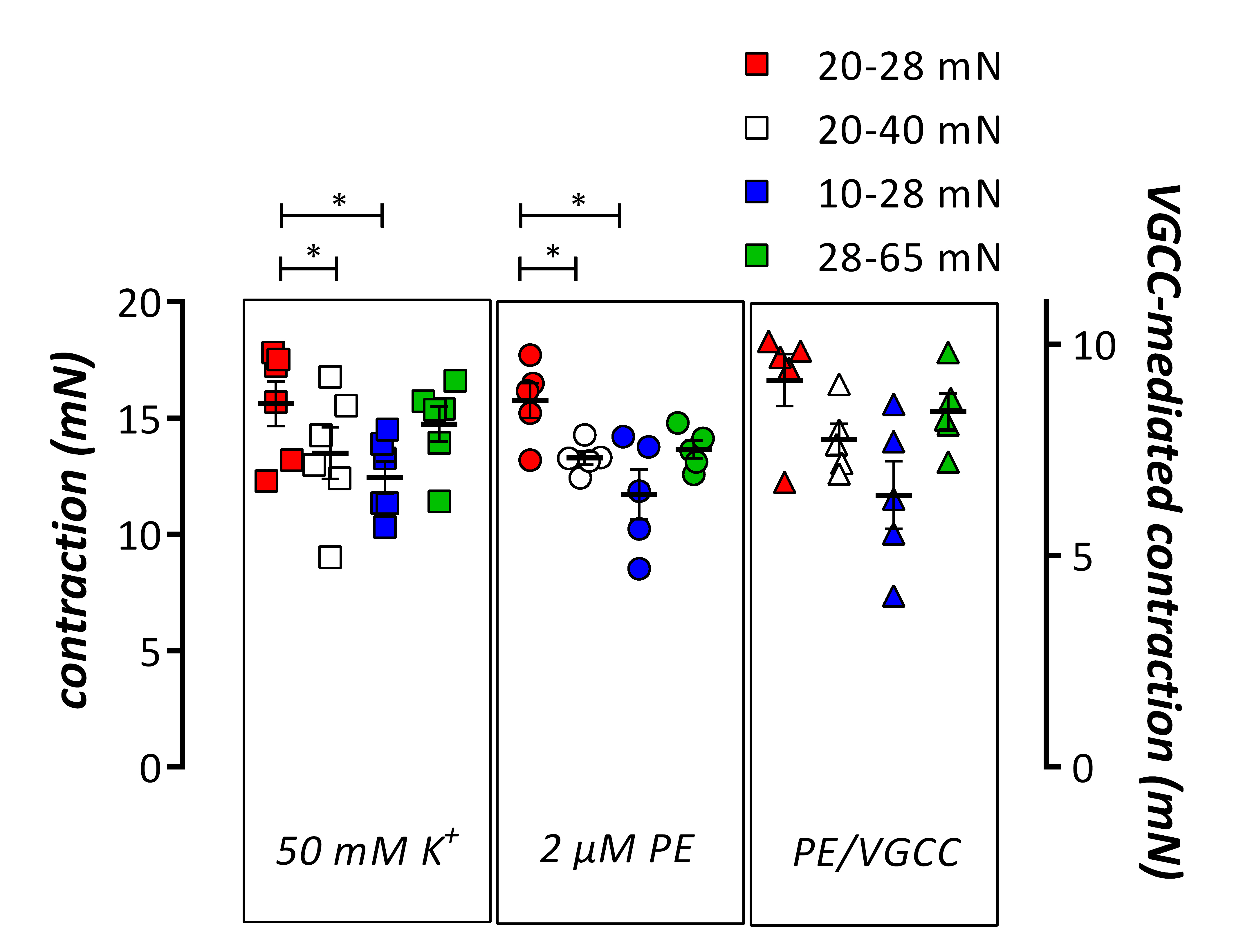

Supplement: Supplementary Figure 3 — In 6 mice we depolarized the segments, which were subjected to conditioning cyclic stretch of 20–28, 20–40, 10–28, and 28–65 mN, and measured the isometric contraction induced by 50 mM external K+. We compared this contraction with the isometric contraction by 2 μM PE in the absence and presence of 35 μM diltiazem to reveal the relative contribution of VGCC to the PE-induced contraction. From the figure it is clear that the VGCC-mediated contraction in the different conditions (triangles, right Y-axis) was dependent on the conditioning cyclic stretch in exactly the same way as the contraction mediated by depolarization with 50 mM K+ (squares, left y-axis). Contractions were somewhat larger than the contractions shown in the submitted manuscript, because the mice were older (8 months), but the overall data were qualitatively the same. n = 6, one way ANOVA with Tukey's multiple comparison test. [file Image3.JPEG]
